# Statistical analyses of ordinal outcomes in randomised controlled trials: a scoping review

**DOI:** 10.1186/s13063-024-08072-2

**Published:** 2024-04-06

**Authors:** Chris J. Selman, Katherine J. Lee, Kristin N. Ferguson, Clare L. Whitehead, Brett J. Manley, Robert K. Mahar

**Affiliations:** 1 Clinical Epidemiology and Biostatistics Unit, Murdoch Children’s Research Institute, Parkville, VIC 3052 Australia; 2 Department of Paediatrics, University of Melbourne, Parkville, VIC 3052 Australia; 3 Centre for Epidemiology and Biostatistics, Melbourne School of Population and Global Health, University of Melbourne, Parkville, VIC 3052 Australia; 4 Department of Obstetrics and Gynaecology, University of Melbourne, Parkville, VIC 3052 Australia; 5 Department of Maternal Fetal Medicine, The Royal Women’s Hospital, Parkville, VIC 3052 Australia; 6 Newborn Research, The Royal Women’s Hospital, Parkville, VIC 3052 Australia; 7 Clinical Sciences, Murdoch Children’s Research Institute, Parkville, VIC 3052 Australia

**Keywords:** Ordinal outcomes, Proportional odds model, Randomised controlled trials, Scoping review

## Abstract

**Background:**

Randomised controlled trials (RCTs) aim to estimate the causal effect of one or more interventions relative to a control. One type of outcome that can be of interest in an RCT is an ordinal outcome, which is useful to answer clinical questions regarding complex and evolving patient states. The target parameter of interest for an ordinal outcome depends on the research question and the assumptions the analyst is willing to make. This review aimed to provide an overview of how ordinal outcomes have been used and analysed in RCTs.

**Methods:**

The review included RCTs with an ordinal primary or secondary outcome published between 2017 and 2022 in four highly ranked medical journals (the *British Medical Journal*, *New England Journal of Medicine*, *The Lancet*, and the *Journal of the American Medical Association*) identified through PubMed. Details regarding the study setting, design, the target parameter, and statistical methods used to analyse the ordinal outcome were extracted.

**Results:**

The search identified 309 studies, of which 144 were eligible for inclusion. The most used target parameter was an odds ratio, reported in 78 (54%) studies. The ordinal outcome was dichotomised for analysis in 47 ($33\%$) studies, and the most common statistical model used to analyse the ordinal outcome on the full ordinal scale was the proportional odds model (64 [$44\%$] studies). Notably, 86 (60%) studies did not explicitly check or describe the robustness of the assumptions for the statistical method(s) used.

**Conclusions:**

The results of this review indicate that in RCTs that use an ordinal outcome, there is variation in the target parameter and the analytical approaches used, with many dichotomising the ordinal outcome. Few studies provided assurance regarding the appropriateness of the assumptions and methods used to analyse the ordinal outcome. More guidance is needed to improve the transparent reporting of the analysis of ordinal outcomes in future trials.

**Supplementary Information:**

The online version contains supplementary material available at 10.1186/s13063-024-08072-2.

## Background

Randomised controlled trials (RCTs) aim to estimate the causal effect of one or more interventions relative to a control or reference intervention. Ordinal outcomes are useful in RCTs because the categories can represent multiple patient states within a single endpoint. The definition of an ordinal outcome is one that comprises monotonically ranked categories that are ordered hierarchically such that the distance between any two categories is not necessarily equal (or even meaningfully quantifiable) [1]. Ordinal outcomes should have categories that are mutually exclusive and unambiguously defined and can be used to capture improvement and deterioration relative to a baseline value where relevant [2]. If an ordinal scale is being used to capture change in patient status, then the ordinal outcome should also be symmetric to avoid favouring a better or worse health outcome [2]. Commonly used ordinal outcomes in RCTs include the modified-Rankin scale, a 7-category measure of disability following stroke or neurological insult [3–6], the Glasgow Outcome Scale-Extended (GOS-E), an 8-category measure of functional impairment post traumatic brain injury [7], and the World Health Organization (WHO) COVID-19 Clinical Progression Scale [8], an 11-point measure of disease severity among patients with COVID-19. The WHO Clinical Progression Scale, developed specifically for COVID-19 in 2020 [8], has been used in many RCTs evaluating COVID-19 disease severity and progression [9, 10] and has helped to increase the familiarity of ordinal data and modelling approaches for ordinal outcomes for clinicians and statisticians alike [11].

Randomised controlled trials that use ordinal outcomes need to be designed and analysed with care. This includes the need to explicitly define the target parameter to compare the intervention groups (i.e. the target of estimation, for example, a proportional odds ratio (OR)), the analysis approach, and whether assumptions used in the analysis are valid. Although this is true for all RCTs, these issues are more complex when using an ordinal outcome compared to a binary or continuous outcome. For example, the choice of target parameter for an ordinal outcome depends on both the research question [12, 13] and the assumptions that the analyst is willing to make about the data.

One option is to preserve the ordinal nature of the outcome, which can give rise to a number of different target parameters. Principled analysis of ordinal data often relies on less familiar statistical methods and underlying assumptions. Many statistical methods have been proposed to analyse ordinal outcomes. One approach to estimate the effect of treatment on the distribution of ordinal endpoints is to use a cumulative logistic model [14, 15]. This model uses the distribution of the cumulative log-odds of the ordinal outcome to estimate a set of ORs [16], which, for an increase in the value of a covariate, represents the odds of being in the same or higher category at each level of the ordinal scale [15]. Modelling is vastly simplified by assuming that each covariate in the model exerts the same effect on the cumulative log odds for each binary split of the ordinal outcome, regardless of the threshold. This is known as the proportional odds (PO) assumption, with the model referred to as ordered logistic regression or the PO model (we refer to the latter term herein). The PO model has desirable properties of palindromic invariance (where the estimates of the parameters are not equivalent when the order of the categories are reversed) and invariance under collapsibility (where the estimated target parameter is changed when categories of the response are combined or removed) [17]. Studies have shown that an ordinal analysis of the outcome using a PO model increases the statistical power relative to an analysis of the dichotomised scale [18, 19]. The target parameter from this model, the proportional or common OR, also has a relatively intuitive interpretation [20, 21], representing a shift in the distribution of ordinal scale scores toward a better outcome in an intervention group compared to a reference group.

The PO model approach makes the assumption that the odds are proportional for each binary split of the ordinal outcome. If this assumption is violated then the proportional OR may be misleading in certain circumstances. Specifically, violation to PO can affect type I or II errors and/or may distort the magnitude of the treatment effect. For example, violation of proportional odds can increase the likelihood of making a type I error since the model may incorrectly identify evidence of a relationship between the treatment and outcome. Violation of the proportional odds assumption may also increase the likelihood of a type II error as the model may fail to identify a relationship between the treatment and the ordinal outcome because the model may fail to capture the true complexity of the relationship. In addition, a treatment may exert a harmful effect for some categories of the ordinal outcome but exert a beneficial effect for the remaining categories, which can ‘average’ out to no treatment effect when assuming a constant OR across the levels of the ordinal scale. The violation of PO may be harmful if the interest is also to estimate predicted probabilities for the categories of the ordinal scale, which will be too low or high for some outcomes when PO is assumed. Although the PO assumption will ‘average’ the treatment effect across the categories of the ordinal outcome, this may not be a problem if all of the treatment effects for each cut-point are in the same direction and the research aim is to simply show whether the treatment is effective even in the presence of non-PO. If the PO assumption is meaningfully violated and the interest is either in the treatment effect on a specific range of the outcome or to obtain predicted probabilities for each category of the scale, the PO model can be extended to a partial proportional odds (PPO) model which allows the PO assumption to be relaxed for a specific set or for all covariates in the model [22]. There are two types of PPO models: the unconstrained PPO model, in which the cumulative log-ORs across each cut-point vary freely across some or all of the cut-points [23], and the constrained PPO model, which assumes some functional relationship between the cumulative log-ORs [21]. However, such an approach may be more inefficient than using a PO model [24, 25].

Alternative statistical methods that can be used to analyse the ordinal outcome include multinomial regression, which estimates an OR for each category of the ordinal outcome relative to the baseline category. The disadvantage of multinomial regression is that the number of ORs requiring estimation increases with the number of categories in the ordinal outcome. A larger sample size may therefore be required to ensure accurate precision of the many target parameters. Other methods are the continuation ratio model or adjacent-category logistic model, though these models lack two desirable properties: palindromic invariance and invariance under collapsibility [15, 17, 26].

Another option is to use alternative methods, such as the Mann-Whitney *U *test or Wilcoxon rank-sum test [27] (referred to as the Wilcoxon test herein). The Wilcoxon test is equivalent to the PO model with a single binary exposure variable [15, 28]. The treatment effect from a Wilcoxon test is the concordance probability that represents the probability that a randomly chosen observation from a treatment group is greater than a randomly chosen observation from a control group [29, 30]. This parameter closely mirrors the OR derived from the PO model. Importantly, the direction of the OR from the PO model always agrees with the direction of the concordance probability. The disadvantages of the Wilcoxon test are that the concordance probability may be unfamiliar to clinicians, and the Wilcoxon test cannot be adjusted for covariates.

Another option is to dichotomise the ordinal outcome and use an OR or risk difference as the target parameter, estimated using logistic or binomial regression. This produces an effect estimate with clear clinical interpretations that may be suitable for specific clinical settings. The disadvantage of dichotomising an ordinal outcome is that it means discarding potentially useful information within the levels of the scale. This means that the trial may require a larger sample size to maintain the same level of statistical power to detect a clinically important treatment effect [19], which may not be feasible in all RCTs depending on cost constraints or the rate of recruitment. The decision to dichotomise may also depend on when the outcome is being measured. This was highlighted in a study that showed that an ordinal analysis of the modified-Rankin scale captured differences in long-term outcomes in survivors of stroke better than an analysis that dichotomised the ordinal outcome [3, 31].

An alternative to dichotomisation is to treat the ordinal outcome as continuous and focus on the mean difference as the target parameter. This choice to treat the outcome as continuous may be based on the number of categories, where the more categories, the more the outcome resembles a continuum if proximate categories measure similar states or if the scale reflects a latent continuous variable. This has the advantage that modelling is straightforward and familiar, but it can lead to ill-defined clinical interpretations of the treatment effect since the difference between proximate categories is unequal nor quantifiable. Such an analysis also wrongly assumes that the outcome has an unbounded range.

### Rationale

There has been commentary [32] and research conducted on the methodology of using ordinal outcomes in certain RCT settings that have mainly focused on the benefit of an ordinal analysis using a PO model [19, 33–35], including investigations into the use of a PPO model when the PO assumption is violated [36]. However, these studies have primarily focused on a limited number of statistical methods and in mostly specific medical areas such as neurology and may not be applicable more generally. Given the growing use of ordinal outcomes in RCTs, it is crucial to gain a deeper understanding of how ordinal outcomes are utilised in practice. This understanding will help identify any issues in the use of ordinal outcomes in RCTs and facilitate discussions on improving the reporting and analysis of such outcomes. To address this, we conducted a scoping review to systematically examine the use and analysis of ordinal outcomes in the current literature. Specifically, we aimed to:

- Identify which target parameters are of interest in RCTs that use an ordinal outcome and whether these are explicitly defined.
- Describe how ordinal outcomes are analysed in RCTs to estimate a treatment effect.
- Describe whether RCTs that use an ordinal outcome adequately report key methodological aspects specific to the analysis of the ordinal outcome.

## Methods

### Protocol

A pre-specified protocol was developed for this scoping review [37]. Deviations from the protocol are outlined in Additional file 1. Here, we provide an overview of the protocol and present the findings from the review which have been reported using the Preferred Reporting Items for Systematic Reviews and Meta-Analyses extension for Scoping Reviews (PRISMA-ScR) checklist [38].

### Eligibility criteria

Studies were included in the review if they were published in one of four highly ranked medical journals (*British Medical Journal* (BMJ), *New England Journal of Medical* (NEJM), *Journal of the American Medical Association* (JAMA), or *The Lancet)* between 1 January 2017 and 31 July 2022 and reported the results of at least one RCT (e.g. if reporting results from multiple trials) with either a primary or secondary outcome that was measured on an ordinal scale. These journals were chosen because they are leading medical journals that publish original and peer-reviewed research with primarily clinical aims and have been used in other reviews of trial methodology [39, 40]. RCTs were defined using the Cochrane definition of an RCT, which is a study that prospectively assigns individuals to one of two (or more) interventions using some random or quasi-random method of allocation [41].

Studies were excluded from this review if they were written in a language other than English, since we did not have sufficient resources to translate studies written in another language. We also excluded studies which were purely methodological, where the abstract or full-text was not available, which reported data from non-human subjects, and those that provided a commentary, review opinion, or were description only. Manuscripts that reported only a trial protocol or statistical analysis plan were also excluded, since one of the main objectives of this review was to determine which statistical methods are being used to analyse trial data. Studies that used ordinal outcomes that were measured on a numerical rating or visual analogue scale were also excluded. Although these scales are often considered ordinal, they imply equidistance between contiguous categories, and can conceivably be analysed as continuous data.

### Information sources

Studies were identified and included in the review by searching the online bibliographic database, PubMed, executed on 3 August, 2022.

### Search strategy

The search strategy for this review was developed by CJS in consultation with KJL and RKM. The search strategy employed terms that have been developed to identify RCTs [41] and terms that have been used to describe an ordinal outcome in published manuscripts for RCTs. The complete search strategy that was used in this review is described in Table 1.

**Table 1:** PubMed search strategy

Search strategy	
(JAMA[journal] OR NEJM[journal] OR lancet[journal] OR BMJ[journal]) AND (ordinal[tiab]^1^ OR categorical[tiab] OR multinomial[tiab] OR “item-response”[tiab] OR psychometric[tiab] OR scale[tiab] OR Likert[tiab]) AND (randomized controlled trial[pt]^2^ OR controlled clinical trial[pt] OR trial[tiab] OR randomized[tiab] OR placebo[tiab] OR clinical trials as topic[mesh: noexp]^3^ OR randomly[tiab])	

^1^ Indicates that the search is conducted on article titles and abstracts only

^2^ Corresponds to a publication type to indicate the article’s type of information conveyed

^3^ Corresponds to a medical subject headings such that the explosion feature has been turned off (explosion searches the more specific terms beneath that heading)

### Selection of sources of evidence

There was no pre-specified sample size for this review. All eligible studies that were identified via the search strategy were included in the review.

Piloting of the eligibility criteria was conducted by CJS and RKM who independently assessed the titles and abstracts of 20 studies to ensure consistency between reviewers. CJS then performed the search on the PubMed database. All titles and abstracts identified were extracted into Covidence, a web-based tool for managing systematic reviews [42]. A two-phase screening process was employed, where all abstracts and titles were screened by CJS in the first phase. Those studies that were not excluded were then moved to the second phase of the screening process, where the full text was evaluated against the eligibility criteria by CJS. A random sample of 40 studies were also assessed for eligibility by a second reviewer (one of KJL, RKM, BJM, or CLW). All studies that were deemed eligible were included in the data extraction.

### Data extraction

A data extraction questionnaire was developed in Covidence [42] and piloted by CJS and RKM using a sample of 10 studies, which was further refined. The final version of the questionnaire is shown in Additional file 2, and a full list of the data extraction items is provided in Table 2. Data was extracted from both the main manuscript and any supplementary material, including statistical analysis plans. CJS extracted data from all eligible studies in the review. Double data extraction was performed by KJL and RKM on a random sample of 20 studies. Any uncertainties in the screening and data extraction process were discussed and resolved by consensus among all reviewers. Simplifications and assumptions that were made for eligibility and data extraction are outlined in Additional file 1.

**Table 2:** Summary of items extracted as part of the review

Category	Extracted data
**Study characteristics**	$\bullet$ Title
	$\bullet$ First author name(s)
	$\bullet$ Publication year
	$\bullet$ Funding source
	$\bullet$ Journal
	$\bullet$ Trial type
**Subject matter**	$\bullet$ Medical condition studied
	$\bullet$ Medical specialty studied
	$\bullet$ Number of study participants included in the analysis (largest if multiple analyses)
**Design**	$\bullet$ Setting
	$\bullet$ Ordinal outcome type
	$\bullet$ Number of categories in the outcome
	$\bullet$ Whether the ordinal outcome was measured at a single time point or as a measure of change
	$\bullet$ Whether the categories of the ordinal scale were clearly defined, ordered, mutually exclusive and, if a measure of change, symmetrical
	$\bullet$ Whether the ordinal outcome was a primary or secondary outcome
	$\bullet$ Whether sample size determination was used based off the ordinal outcome
	$\bullet$ Target parameter used
**Statistical methods**	$\bullet$ The statistical model(s) or method(s) that were used to analyse the ordinal outcome
	$\bullet$ Type of inference used (frequentist/Bayesian)
	$\bullet$ How the distribution of the ordinal outcome was summarised by intervention
	$\bullet$ Methods used to account for repeated measures over time (if applicable)
	$\bullet$ Details on whether the model assumptions were reported
	$\bullet$ Whether the analysis that was reported in the results differed from the analysis outlined in the methods section of the manuscript
**Software included**	$\bullet$ Statistical software package used for the analysis

### Synthesis of results

The data extracted from Covidence were cleaned and analysed using Stata [43]. Descriptive statistics were used to summarise the data. Frequencies and percentages and medians and interquartile ranges (IQRs) were reported for categorical and continuous variables respectively. Qualitative data were synthesised in a narrative format.

## Results

### Results of the search

**Fig. 1:**
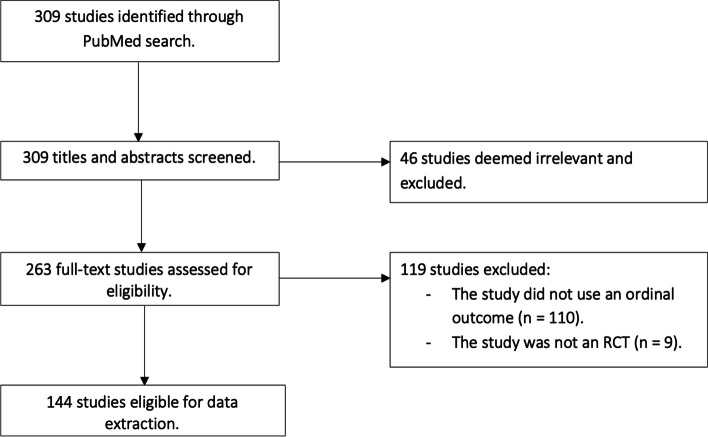
Flow diagram of the study

The initial search identified 309 studies, of which 46 were excluded for not being an RCT. There were 263 studies that underwent full text review. Of these, 119 were excluded: 110 because they did not have an ordinal outcome, and nine because they were not an RCT. In total, 144 studies were eligible for data extraction [44–187]. A flow diagram of the study selection is shown in Fig. 1. The questionnaire that was used to extract the data from each study is provided in Additional file 2.

### Study characteristics

A summary of the study characteristics is presented in Table 3. The highest proportion of studies were published in the *NEJM* (61 studies, $42\%$), followed by *JAMA* (40, 28%) and *The Lancet* (34, 24%), with only nine studies published in the *BMJ* ($6\%$). The number of studies that used an ordinal outcome were higher in 2020 and 2021 ($30, 21\%$ in each year) compared to earlier years ($21, 15\%$ in 2019, $24, 17\%$ in 2018 and $23, 16\%$ in 2017). Nearly all studies were conducted in a clinical setting ($141, 98\%$). The most common medical condition being studied was stroke ($39, 28\%$), followed by COVID-19 ($22, 16\%$) and atopic dermatitis ($6, 4\%$). The most common medical field was neurology ($54, 38\%$) followed by infectious diseases ($22, 16\%$, all of which were COVID-19 studies), dermatology ($13, 9\%$), and psychiatry ($12, 9\%$). Studies were mostly funded by public sources ($104, 72\%$). The median number of participants in the primary analysis of the ordinal outcome was 380 (interquartile range (IQR): 202–803).

**Table 3:** Summary of study characteristics and subject matter

	**Studies―***n*** (%)**
Number of publications	144
Year of publication―*n* (%)	
- 2017	23 (16%)
- 2018	24 (17%)
- 2019	21 (15%)
- 2020	30 (21%)
- 2021	30 (21%)
- 2022	16 (11%)
Funding source/s―*n* (%)^a^	
- Public	104 (72%)
- Industry	57 (40%)
- Non-profit	39 (27%)
Journal―*n* (%)	
- NEJM	61 (42%)
- JAMA	40 (28%)
- The Lancet	34 (24%)
- BMJ	9 (6%)
Medical condition studied - n (%)	
- Stroke	39 (28%)
- COVID-19	22 (16%)
- Atopic dermatitis	6 (4%)
- Cardiac arrest	5 (3%)
- Other	73 (51%)
Medical specialty studied―*n* (%)	
- Neurology	54 (38%)
- Infectious diseases	22 (16%)
- Dermatology	13 (9%)
- Psychiatry	12 (9%)
- Cardiology	7 (5%)
- Rheumatology	7 (5%)
- Other	26 (18%)
Clinical setting―*n* (%)	141 (98%)
Adaptive design used ―*n* (%)^‡^	58 (40%)
Participants in analysis―median (IQR, range)^b^	380 (202–803, 21–11016)

^a^The total does not add up to the total number of publications as more than one option could be selected

^b^The number of participants in the primary analysis could be extracted for 142 studies

^‡^We did not include studies in which the stopping rules were not definitive, e.g. if a study reported that the study might stop early for efficacy if the *p*-value was very small but the ultimate recommendation would be decided by the Data Safety & Monitoring Committee

Of the 144 included studies, 58 (40%) used some form of adaptive design, with 47 ($33\%$) having explicitly defined early stopping rules for efficacy or futility, 18 ($13\%$) used sample size re-estimation, three ($2\%$) used response adaptive randomisation, three ($2\%$) used covariate adaptive randomisation, three ($2\%$) were platform trials, and three ($2\%$) used adaptive enrichment that focused on specific subgroups of patients.

### Ordinal outcomes and target parameters

A summary of the properties of the ordinal outcomes used in the studies is shown in Table 4. An ordinal scale was used as a primary outcome in 59 ($41\%$) of studies. Most studies used an ordinal scale to describe an outcome at a single point in time ($128, 89\%$), with 16 studies using an ordinal outcome to capture changes over time ($11\%$). One study used a Likert scale where the categories were ambiguously defined in the manuscript. Another study used an ordinal outcome to measure change over time, but it was asymmetric and biased towards a favourable outcome. The median number of categories in the ordinal outcome was 7 (IQR: 6–7) and ranged from 3 to 23 categories.

**Table 4:** Summary of the ordinal outcome

Design	Studies―*n* (%)
Type of ordinal scale used―*n* (%)	
- Single-state scale	128 (89%)
- Transition-state scale	16 (11%)
Type of outcome―*n* (%)	
- Primary outcome	59 (41%)
- Secondary outcome	85 (59%)
Type of outcome measure―*n* (%)	
- Clinical outcomes	130 (90%)
- Life impact	11 (8%)
- Physiological outcome	1 (1%)
- Adverse event	1 (1%)
- Other	1 (1%)
Target parameter(s)―*n* (%)^a^	
- Odds ratio	78 (54%)
- Risk difference	31 (22%)
- Non-parametric procedure used	19 (13%)
- Risk ratio	13 (9%)
- Difference in means	11 (8%)
- Difference in medians	8 (6%)
- Other	8 (6%)
- Descriptive analysis only	6 (4%)
- Unknown	4 (3%)
*A priori* sample size based on the ordinal outcome―*n* (%)^a^	32
- Analytical	26 (81%)
- Simulation^†^	6 (19%)
Scale dichotomised for analysis―*n* (%)	47 (33%)
No. of categories in ordinal outcome―median (IQR, range)	7 (6–7; 3–23)

^a^The sample size (*N* = 98) here is not the same as the number of studies that analysed using an ordinal scale since one study planned to analyse the ordinal outcome in its original scale, but the assumptions of the statistical model were violated and was thus dichotomised for analysis. We retained a sample size of *N* = 98 instead of focussing on primary outcomes only as the sample size was determined from the secondary ordinal outcome in one study. There were 32 studies that estimated the sample size based on the ordinal outcome

^†^Of those studies that used simulation to determine the sample size, four of these studies used an adaptive design (either early stopping or adaptive enrichment); four studies used frequentist inference, one used Bayesian inference alone and another study used a combination of both

There were 32 studies that determined the sample size in advance based on the ordinal outcome, of which 26 out of 32 studies ($81\%$) used an analytical approach and 6 out of 32 studies ($19\%$) used simulation to estimate the sample size. Among those studies that used an analytical approach, five studies reported to have used the Whitehead method and three studies reported to have used a *t*-test. Among the remaining studies that used an analytical approach, it was unclear which specific method was used to compute the sample size.

The ordinal outcome was dichotomised for analysis in 47 ($33\%$) studies. Some justifications for the dichotomisation of the ordinal outcome included that it represented a clinically meaningful effect and/or that it was common in the analysis of the outcome in similar studies (reported in 24 studies), that the dichotomised outcome represented an agreeable endpoint based on feedback between clinicians and/or patients and families (two studies), or that the assumptions of the statistical model for the categorical outcome were violated (reported in three studies).

There were a variety of target parameters used for the ordinal outcomes. In 130 studies, the target parameter could be determined; however, 59 of these studies ($45\%$) did not clearly or explicitly define the target parameter of interest. Of those where the target parameter could be determined based on the information provided in the manuscript (e.g. since it was not reported), an OR was the most common target parameter ($78, 54\%$), followed by a risk difference ($31, 22\%$). A difference in mean or median was the target parameter in 11 (8%) and 8 (6%) studies respectively. There were 14 ($10\%$) studies that did not estimate a target parameter. This was either because the study was descriptive in nature, the analysis used a non-parametric procedure, or the target parameter could not be determined (or some combination thereof).

### Statistical methods and assumptions

There was a variety of descriptive measures used to summarise the distribution of the ordinal outcome by intervention groups (Table 5). The most common descriptive statistics were frequencies and/or percentages in each category of the ordinal outcome ($116, 81\%$), followed by the median score across all categories ($33, 23\%$) and IQRs ($31, 22\%$). The mean and standard deviation across the categories of the ordinal outcome were only summarised in 16 (11%) and 10 (7%) studies respectively.

**Table 5:** Summary of the statistical methods used in the analysis of the ordinal outcome

Statistical methods	Studies―*n* (%)
Descriptive statistics―*n* (%)^a^	
- Frequencies and/or proportions/percentages (category specific)	116 (81%)
- Medians (across all categories)	33 (23%)
- Interquartile ranges (across all categories)	31 (22%)
- Means (across all categories)	16 (11%)
- Standard deviations (across all categories)	10 (7%)
- Other methods to summarise by group used	10 (7%)
- Summaries by group not used	2 (1%)
Inferential approach―*n* (%)	
- Frequentist inference	131 (91%)
- Bayesian inference	6 (4%)
- Both frequentist and Bayesian	2 (1%)
- Descriptive analysis only	5 (4%)
Statistical method(s)―*n* (%)^a^	
- Proportional odds model	64 (44%)
- Logistic regression model	16 (11%)
- Linear model	16 (11%)
- Cochran-Mantel-Haenszel test	15 (10%)
- Wilcoxon test	14 (10%)
- Fisher’s exact test or chi-square test	12 (8%)
- Binomial regression model	7 (5%)
- Other	24 (17%)
- Could not be determined	6 (4%)
- Descriptive analysis only	6 (4%)
Original statistical method(s) modified―*n* (%)^b^	
- No	124 (86%)
- Yes	12 (8%)
- Unknown	2 (1%)
- Not applicable	6 (4%)
Statistical method(s) assumptions checked and clearly described―*n* (%)	
- No	86 (60%)
- Yes	46 (32%)
- Unknown	5 (4%)
- Not applicable (descriptive analysis or only bootstrapping methods used)	7 (5%)
Methods used to check assumptions:^a^	46
- Statistical methods	31 (67%)
- Graphical methods	2 (4%)
- Prediction methods	1 (2%)
- Other	7 (15%)
- Not reported	6 (13%)
Methods used to account for repeated measures―*n* (%)^a^	38
- Adjusted for baseline measurement	18 (47%)
- Mixed effects models	14 (37%)
- Generalised estimating equations	4 (11%)
- Other	4 (11%)
- Unknown	2 (5%)
Software package(s) used―*n* (%)^a^	
- SAS	81 (56%)
- R	35 (24%)
- Stata	27 (19%)
- SPSS	13 (9%)
- Other	9 (6%)
- Unknown	12 (8%)

^a^The total does not add up to the total number of publications as more than one option could be selected

^b^Different statistical model/method could also mean using the same model to analyse the ordinal outcome (e.g. from the cumulative probability model family) but removing covariates to ensure the model assumptions are met (e.g. the proportional odds assumption)

Many different statistical methods were used to analyse the ordinal outcome (Table 5). The PO model was the most common statistical method used to analyse the ordinal outcome (64, $44\%$) that was used to estimate a proportional OR in 62 studies. In studies that used a PO model for the analysis, the interpretation of the target parameter varied between studies (see Additional file 3). The most frequent definition used was that the proportional OR represented an ordinal shift in the distribution of ordinal scale scores toward a better outcome in the intervention relative to the control group ($12, 19\%$). When the outcome was dichotomised, logistic regression was used in 16 studies ($11\%$ of all studies) that usually estimated an OR or a risk difference using g-computation. Seven studies estimated a risk difference or risk ratio using binomial regression. Studies also calculated and reported a risk difference with corresponding $95\%$ confidence intervals estimated using methods such as the Wald method or bootstrapping ($31, 22\%$). There were 19 (13%) studies that used a non-parametric method to analyse the ordinal outcome (either dichotomised or not), including the Cochran-Mantel-Haenszel test ($15, 10\%$) to estimate an odds ratio, the Wilcoxon test ($14, 10\%$), of which no study reported a concordance probability as the target parameter, or the Fisher’s exact or Chi-Square test (12, $8\%$). Other statistical methods that were used were the Hodges-Lehmann estimator, used to estimate a median difference ($3, 2\%$) and the Van Elteren test ($2, 1\%$), an extension of the Wilcoxon test for comparing treatments in a stratified experiment. Linear regression was used in 16 ($11\%$) studies that tended to estimate a mean or risk difference (despite the model having an unbounded support).

The majority of studies ($86, 60\%$) did not explicitly check the validity of the assumptions for the statistical method(s) used. For example, no study that analysed the ordinal outcome using linear regression commented on the appropriateness of assigning specific numbers of the outcome categories. Among the 64 studies that used a PO model, 20 (31%) did not report whether the assumption of PO was satisfied. Overall, there were 46 studies that reported checking key modelling assumptions; however, the method that was used to check these assumptions were not reported in 6 ($13\%)$ of these studies. The most common way to verify model assumptions was to use statistical methods ($31, 67\%$), followed by graphical methods ($2, 4\%$).

Among the 44 studies that assessed the validity of the PO assumption for a PO model, 13 studies ($30\%$) used a likelihood ratio test, 10 studies ($23\%$) used the Brant test, and 10 studies ($23\%$) also used the Score test. Six ($14\%$) studies assessed the robustness of the PO assumption by fitting a logistic regression model to every level of the ordinal outcome across the scale, in which the OR for each dichotomous break was presented. Two studies assessed the PO assumption using graphical methods, which plotted either the inverse cumulative log odds or the empirical cumulative log odds. It was unclear which method was used to assess the PO assumption in ten studies that reported to have checked the assumption.

There were 12 studies ($8\%$) that reported using a different statistical method than originally planned. Ten of these studies had originally planned to use a PO model, but the PO assumption was determined to have been violated and an alternative method was chosen. One study removed the covariate that was reported to have violated the PO assumption and still used a PO model to analyse the outcome. Two studies used an unconstrained PPO model that reported an adjusted OR for each binary split of the ordinal outcome. Three studies used a Wilcoxon test, with one study stratifying by a baseline covariate that violated the PO assumption. Another study dichotomised the ordinal outcome for the analysis. One study used a Van Elteren test that estimated a median difference (which inappropriately assumes that there is an equal distance between proximate categories), another used a Poisson model with robust standard errors, and one study retained the analysis despite the violation in PO. Notably, a PPO model was not reported to have been used in studies that reported that a covariate other than the treatment violated the PO assumption. Seven studies also did not report which covariate(s) violated the PO assumption.

Frequentist inference was the most common framework for conducting the analysis (133, 92%), with Bayesian methods being used in eight (6%) studies (where two studies used both), of which all eight studies used an adaptive design. Of those using Bayesian methods, seven studies used a Bayesian PO model for analysis. Of these studies, four used a Dirichlet prior distribution to model the baseline probabilities, and three used a normally distributed prior on the proportional log-OR scale. Two of these studies reported to use the median proportional OR with corresponding $95\%$ credible interval, while one study reported the mean proportional OR. Three studies reported that the models were fitted with the use of a Markov-chain Monte Carlo algorithm with either 10, 000 (one study) or 100, 000 (two studies) samples from the joint posterior distribution. No study reported how the goodness-of-fit of the model was assessed.

For the 38 studies that collected repeated measurements on the ordinal outcome, 18 adjusted for the baseline measurement ($47\%$), 14 used mixed effects models ($37\%$), and four used generalised estimated equations ($11\%$) to capture the correlation among the repeated measures for an individual.

A range of statistical packages were used for the analysis of the ordinal outcome, with SAS ($81, 56\%$) and R ($35, 24\%$) being most common. Twelve ($8\%$) studies did not report the software used.

## Discussion

This review has provided an overview of how ordinal outcomes are used and analysed in contemporary RCTs. We describe the insight this review has provided on the study design, statistical analyses and reporting of trials using ordinal outcomes.

### Target parameter

The target parameter of interest is an important consideration when planning any trial and should be aligned with the research question [12, 13]. The most common target parameter in this review was an OR, either for a dichotomised version of the ordinal outcome or in an analysis that used the ordinal scale. When an ordinal analysis was used, it was common that the target parameter was a proportional OR, although there was variation in the interpretation of this parameter between studies. We found that it was most common to interpret the proportional OR as an average shift in the distribution of the ordinal scale scores toward a better outcome in the intervention, relative to the comparator(s) [19, 35, 188, 189]. In the studies that dichotomised the ordinal outcome, many lacked justification for doing so and, in one case, dichotomisation was used only due to the violation of PO, despite the fact that this changed the target parameter.

Some studies in our review treated the ordinal outcome as if it were continuous, and used a difference in means or medians as the target parameter. These quantities do not represent a clinically meaningful effect when the outcome is ordinal, since proximate categories in the scale are not necessarily separated by a quantifiable or equal distance, which can affect the translation of the trial results into practice. If a study is to use a mean difference then the researchers should justify the appropriateness of assigning specific numbers used to the ordinal outcome categories.

The target parameter and statistical method used to estimate it could not be determined in some studies. Notably, the definition of the target parameter was not explicitly defined in almost half of the studies, despite the current recommendations on the importance of clearly defining the estimand of interest, one component of which is the target parameter [12, 13]. Furthermore, there is a lack of clarity in defining the target parameter when a PO model was used, despite the interpretation being analogous to the OR for a binary outcome, but applying to an interval of the ordinal scale instead of a single value. Consistency in the definition of a target parameter in RCTs can allow easy interpretation for clinicians and applied researchers. Explicit definition of the target parameter of interest is essential for readers to understand the interpretation of a clinically meaningful treatment effect, and also reflects the present push within clinical research with regards to estimands [12, 13].

### Statistical methods

It is important to summarise the distribution of the outcome by intervention group in any RCT. When the outcome is ordinal, frequencies and percentages in each category can provide a useful summary of this distribution. Most studies in this review reported frequencies and percentages in each category, although some studies that dichotomised the outcome only reported these summaries for the dichotomised scale. Some studies reported means and standard deviations across the categories which, as mentioned previously, may not have a valid interpretation.

Although there are a range of statistical methods that can be used to analyse an ordinal outcome, we found that the PO model was the most commonly used. This is likely because the PO model is relatively well-known among statisticians and is quite straightforward to fit in most statistical packages, and it possesses the desirable properties of palindromic invariance and invariance under collapsibility. However, when using this approach to estimate a specific treatment effect across all levels of the outcome, it is important to assess and report whether the PO assumption has been met when the aim is to estimate the treatment effect across the different categories or to estimate predicted probabilities in each category. The validity of the PO assumption is less important when the objective is to understand whether one treatment is ‘better’ on average compared to a comparator. In this review, it was common for studies that used a PO model to define the target parameter that related to a treatment benefiting patients with regard to every level of the outcome scale. However, only 44 out of 64 studies reported to have checked the PO assumption, which highlights the deficiency in this practice. Statistical methods were commonly used to assess the PO assumption, although it may be preferable to avoid hypothesis testing when assessing the PO assumption, particularly with small sample sizes, as these statistical tests can have poor statistical power [22, 190]. Also, researchers should keep in mind that when the PO assumption is tested, the type I error of the analysis may change and that *p*-values and confidence intervals based on the updated model ignore the model-fitting uncertainty [191].

When the PO assumption was violated, a PPO model was rarely used, and instead baseline covariates were removed from the model to address the departure to PO. The fact that the PPO model is underused could be due to a lack of knowledge that such models exist and can be used to address violations in PO. Such a model could have been particularly useful in these studies that had only covariates other than the treatment variable that violated the PO assumption, as the PPO model could have been used to estimate a single proportional OR for the treatment effect. Of note, however, is that an unconstrained PPO model does not necessarily require ordinality as the categories can be arranged and the model fit would be hardly affected [192], and that estimated probabilities can be negative [193].

There are other methods that can be used to assess the validity of the PO assumption, such as plotting the differences in predicted log-odds between different categories of the ordinal outcome that should be parallel [16]. Another option is to fit a logistic regression model to every level of the ordinal outcome across the scale and compare the estimated ORs and corresponding confidence interval for each binary split of the ordinal outcome or simulating predictive distributions. However, estimating separate ORs in this way can be inefficient, particularly when the ordinal outcome has a high number of categories. Arguably, more important than assessing the validity of the PO assumption is to assess the impact of making compared to not making the assumption. If the treatment effect goes in the same direction across each category of the ordinal scale and the objective is to simply understand whether one treatment is better overall, then departures from PO may not be important. If, however, the interest is in estimating a treatment effect for every level of the ordinal outcome and/or the treatment has a detrimental effect for one end of the ordinal scale but a beneficial effect for the remaining categories, there should be careful consideration as to the validity to the type I and II error and the treatment effect if the PO model is used.

Finally, a handful of studies also used the Wilcoxon, Chi-Square, or Fisher’s exact test (the latter being too conservative [194] and potentially providing misleading results), where commonly only a *p*-value, not a target parameter, was reported when these methods were used. The lack of a target parameter for the treatment effect can make it difficult for clinicians to translate the results to practice.

### Strengths and limitations

The strengths of this study are that we present a review of a large number of RCTs that used ordinal outcomes published in four highly ranked medical journals to highlight the current state of practice for analysing ordinal outcomes. The screening and data extraction process was conducted systematically, and pilot tests and double data extraction ensured the consistency and reliability of the extracted data. The PRISMA-ScR checklist was used to ensure that reporting has been conducted to the highest standard.

This review does, however, have limitations. The restriction to the PubMed database and four highly ranked medical journals may affect the generalisability of this review. We made this decision given the scoping nature of the review, to ensure reproducibility and to ensure that the total number of studies included in the review was manageable. We also aimed to include studies that are likely to reflect best practice of how research using ordinal outcomes is being conducted and reported upon at present. Given the selected journals represent highly ranked medical journals, these findings are likely to reflect the best-case scenario given these journals' reputation for rigour. In addition, our search strategy may have missed certain phrases or variants (particularly related to an ordinal outcome); however, we attempted to mitigate this through our piloting phase. Finally, we also did not review the protocol papers of the trials that may have included additional information related to the statistical methodology. This includes methods that were planned to be used to assess the PO assumption, and any alternative methods that were to be used instead.

### Implications of this research

This review has implications for researchers designing RCTs that use an ordinal outcome. Although the majority of studies included in this review were in the fields of neurology and infectious diseases, the results of this review would apply to RCTs in all medical fields that use an ordinal outcome. We have shown that there is substantial variation in the analysis and reporting of ordinal outcomes in practice. Our results suggest that researchers should carefully consider the target parameter of interest and explicitly report what the target parameter represents; this is particularly important for an ordinal outcome which can be unfamiliar to readers. Defining the target parameter upfront will help to ensure that appropriate analytical methods are used to analyse the ordinal outcome and make transparent the assumptions the researchers are willing to make.

Our review also highlights the need for careful assessment and reporting of the validity of the model assumptions made during the analysis of an ordinal outcome. Doing so will ensure that robust statistical methods that align with the research question and categorical nature of the ordinal outcome are used to estimate a valid, clinically relevant target parameter that can be translated to practice.

### Supplementary information

**Additional file 1.** Deviations from the protocol. This presents a summary of the deviations from the protocol, with reasons. We also provide an explanation of any simplifications and assumptions that were made for eligibility criteria and data extraction.

**Additional file 2.** Data extraction questionnaire. This is a copy of the data extraction questionnaire that will be used for this review in PDF format.

**Additional file 3.** Interpretation of the proportional odds ratio in proportional odds models. This presents a summary of the ways that the proportional odds ratio was interpreted across the studies.

## Data Availability

The datasets and code generated and/or analysed during the current study are available on GitHub [195].

## Abbreviations

RCT: Randomised controlled trial
PO: Proportional odds
PPO: Partial proportional odds
SAP: Statistical analysis plan
