# Supplementary material for: Statistical analyses of ordinal outcomes in randomised controlled trials: a scoping review
Source: Trials. 2024 Apr 6;25:241. doi: 10.1186/s13063-024-08072-2 (PMC10998402; doi:10.1186/s13063-024-08072-2)
Supplement: Supplementary file 1 — Additional file 1. Deviations from the protocol. This presents a summary of the deviations from the protocol, with reasons. We also provide an explanation of any simplifications and assumptions that were made for eligibility criteria and data extraction. [file 13063_2024_8072_MOESM1_ESM.pdf]

Additional File 1

Deviations from the protocol

There was only one deviation from the protocol. We originally planned to consider any studies published between 1 January 2012 to 31 July 2022 to be eligible for the review. However, this search resulted in 258 eligible studies for data extraction. Due to the amount of time and resources that would be needed to extract this number of studies, we deemed it was appropriate to restrict the time of publication to 1 January 2017 to 31 July 2022 to ensure the amount of data extraction was manageable. With this restriction, 144 studies were eligible for data extraction.

Simplifications and assumptions

The simplifications and assumptions made for eligibility criteria and data extraction include the following:

- The ordinal scale must have 3 or more categories.
- We considered tertiary or exploratory ordinal outcomes in the case where the authors emphasised and reported the analysis of such outcomes in the manuscript.
- We considered adverse event outcomes to be ordinal only if they were explicitly used as a primary or secondary outcome, rather than a safety outcome.
- Details regarding the sample size were not extracted if the sample size was calculated using a dichotomised version of the ordinal scale.
- If an RCT reported to have used rules for early stopping, we considered an RCT to have been adaptive only if these rules for stopping early were strict and based off pre-defined values (such as p-values or posterior probabilities), and not solely based off a ‘possible’ recommendation from the Data Safety and Monitoring Committee.
- It was assumed that the statistical methods reported in the manuscript, unless otherwise unclear, were the only methods that were used to analyse the ordinal outcome.
- If it was difficult to extract a precise definition of the target parameter, the interpretation of the treatment effect that involved the ordinal outcome was extracted which still assisted with the synthesis of the free text in the analysis.
- We extracted data for analyses that were post-hoc only if the scale was analysed on the original ordinal scale, and is reported as an additional (tertiary or exploratory) outcome. For example, a study that categorises a continuous endpoint into an ordinal outcome for a post-hoc analysis to compare statistical methods was not considered to be eligible.
- We only report the number of categories the ordinal scale had prior to any analysis (including prior to dichotomisation, if applicable).
- If a study reported on two (or more) ordinal outcomes where at least one outcome has been dichotomised, we only extracted data for the first outcome that was mentioned **and** analysed on the full ordinal scale.
- If the trial was a platform trial, we extracted data from all relevant studies that were included in the search strategy.

- If there were multiple trials included in the same manuscript but were similar to each other (e.g. the results of multiple trials were pooled), then the first trial that is mentioned and used an ordinal outcome will be included in the review.
- If there were multiple ordinal outcomes, we will only extract data for the first ordinal outcome mentioned in the manuscript.
- Although there could arguably be many medical specialties that the study could focus on, we selected the field that seemed most appropriate, which was done in consultation with clinicians.
