# Supplementary material for: Statistical analyses of ordinal outcomes in randomised controlled trials: a scoping review
Source: Trials. 2024 Apr 6;25:241. doi: 10.1186/s13063-024-08072-2 (PMC10998402; doi:10.1186/s13063-024-08072-2)
Supplement: Supplementary file 3 — Additional file 3. Interpretation of the proportional odds ratio in proportional odds models. This presents a summary of the ways that the proportional odds ratio was interpreted across the studies. [file 13063_2024_8072_MOESM3_ESM.pdf]

Additional File 3

Interpretation of the proportional odds ratio in cumulative logit models

The most common ways to define a proportional odds ratio in studies that analysed the ordinal outcome using a cumulative logistic regression model were as follows:

- An ordinal shift across the range/in the distribution of ordinal scale scores toward a better outcome in the intervention relative to the control group ($n = 12$).
- Odds ratio for a one unit increase in the ordinal scale for intervention compared to the control group ($n = 6$).
- Differences regarding the full distribution of the ordinal scale ($n = 6$).
- An odds ratio greater than 1.0 on the ordinal scale corresponds to more favourable outcomes with the use of treatment as compared with control ($n = 6$).
- The ratio of the cumulative odds of being in a better category of the ordinal outcome for the intervention compared to the control group ($n = 5$).
- The proportional odds ratio of the shift analysis on the ordinal scale ($n = 3$).
- A proportional effect of treatment across the scale of the ordinal outcome ($n = 3$).
- Odds ratio of a better clinical status distribution on the ordinal scale compared between intervention and control groups ($n = 2$).
- Between group differences in the proportional odds of having a higher (worse) score on the ordinal scale ($n = 1$).
